# Effects of Zinc Oxide Nanoparticles (ZnO NPs) on Growth, Immune Responses and Histopathological Alterations in Asian Seabass (*Lates calcarifer*, Bloch 1790) under Low-Salinity Conditions

**DOI:** 10.3390/ani14182737

**Published:** 2024-09-21

**Authors:** Roochira Sukhsangchan, Jitraporn Phaksopa, Anurak Uchuwittayakul, Chi-Chung Chou, Prapansak Srisapoome

**Affiliations:** 1 Laboratory of Aquatic Animal Health Management, Department of Aquaculture, Faculty of Fisheries, Kasetsart University, Bangkok 10900, Thailand; roochira682@gmail.com (R.S.); ffisarb@ku.ac.th (A.U.); 2 Center of Excellence in Aquatic Animal Health Management (CE-AAHM), Faculty of Fisheries, Kasetsart University, Bangkok 10900, Thailand; 3 Department of Marine Science, Faculty of Fisheries, Kasetsart University, Bangkok 10900, Thailand; jitraporn.p@ku.th (J.P.); ccchou@nchu.edu.tw (C.-C.C.); 4 Department of Veterinary Medicine, College of Veterinary Medicine, National Chung Hsing University, Taichung 402, Taiwan

**Keywords:** zinc oxide nanoparticles, Asian seabass, growth, gene expression, hematological alteration, immunotoxicology

## Abstract

**Simple Summary:**

Zinc oxide nanoparticles (ZnO NPs) are versatile chemicals that are widely used in various industries. In this study, the toxic effects of ZnO NPs were thoroughly investigated on Asian seabass, a polyhaline fish species that is now commercially farmed in low-salinity areas of Thailand. Asian seabass fingerlings were used as an animal model and exposed to aqueous solutions of 1–50 ppm ZnO NPs for 8 weeks. During this period, growth parameters, immune parameters and immune-related gene suppression were monitored. Water quality, histopathological alterations and Zn concentrations were also measured. Compared with the control, all ZnO NP concentrations severely inhibited growth and reduced serum levels of innate immune factors. Additionally, the highest concentration of ZnO NPs significantly decreased the expression levels of both innate and adaptive immune-related genes early in the exposure and strongly increased the mortality rate (100%) by week 6. The obtained results provide crucial toxicological data for determining the impact of ZnO NPs on Asian seabass aquaculture, particularly in industrial areas with a high contamination risk, and for assessing the biosecurity of fish consumers.

**Abstract:**

In the present study, Asian seabass (*Lates calcarifer*, Bloch) fingerings were used as an animal model to investigate the toxicological effects of zinc oxide nanoparticles (ZnO NPs) under 5 ppt estuarine conditions. The fish were exposed to 0, 1, 5 or 50 ppm ZnO NPs for 8 weeks. It was found that ZnO NP concentrations of 5–50 ppm negatively affected several growth rate parameters, such as the weight and total length of the fish. Additionally, 5 and 50 ppm ZnO NPs led to 32.55% and 100% mortality, respectively, after 8 weeks after exposure (WAE). Furthermore, compared with the control, exposure to 1–50 ppm ZnO NPs strongly affected hematological indices, such as total blood cells, red blood cells, leukocytes and hematocrit, and suppressed lysozyme activity, superoxide anion production and bactericidal activity. High Zn concentrations accumulated in the head kidney, gills and liver, whereas low levels were detected in the gut, skin and muscle. Expression analysis of immune-related genes via quantitative real-time RT-PCR revealed that 5 and 50 ppm ZnO NPs significantly upregulated the *cc* and *cd4* genes at 1 WAE. In contrast, 50 ppm ZnNPs downregulated the expression levels of the *cd8*, *cc*, *hsp70*, *hsp90*, *tcrα*, *lyz* and *igmh* genes at 1 WAE (*p* < 0.05). Finally, at 8 WAE, histopathological analysis revealed that 5 and 50 ppm ZnO NPs severely induced alterations in the head kidney, gills and liver.

## 1. Introduction

Over the past decade, nanotechnology has become a fascinating topic in nearly all fields, from research to production and application. Nanotechnology, an up-and-coming technology that spans science and technological applications [1,2], emerged from the convergence of physics, chemistry and biology as sciences, leading to the development of various materials, including nanoparticles (NPs) with at least one dimension under 100 nm [3,4]. Among these materials, zinc oxide nanoparticles (ZnO NPs), as crucial metal oxide nanoparticles, are popularly employed in various fields; their unique physical and chemical properties, such as high electrochemical coupling coefficients, high photostability, high chemical stability and a broad range of radiation absorption, make them multifunctional materials [5,6,7]. In particular, ZnO is an essential inorganic pigment in the rubber industry because of its excellent properties as a catalyst for sulfur vulcanization. The tire industry remains the largest market for ZnO, accounting for more than half of the total global ZnO consumption [8,9]. In addition, ZnO is often used as an ingredient in sunscreens in the pharmaceutical and cosmetic industries [10], as well as in the paint and pigment industries [11], the electronics and electrotechnology industries, sensors, photoelectronic devices, photocatalysts and agriculture [12,13].

At present, ZnO NPs are highly persistent in the environment because of natural processes, human effects (e.g., precipitation of nanometer-scale metal oxides in acid mine drainage) and the food chain. This environmental persistence represents an essential pathway for investigating the intake of ZnO NPs to high-trophic-level organisms, making them very dangerous to the environment and aquatic organisms [14,15,16,17]. ZnO NPs are known to have a wide range of harmful effects (e.g., inducing oxidative stress, DNA damage, mutations, reduced cell viability and stimulation of apoptosis and necrosis) on living organisms and interact with immune compartments, resulting in immunosuppression [18,19].

ZnO NPs affect a wide range of aquatic animals. Studies have investigated the impact of ZnO NPs on the physiological performance, immune responses and survival of marine mussels (*Mytilus coruscus*) [20,21]. In fish populations, ecological changes and degradation of natural spawning grounds in most water bodies are leading to rapid decreases in the numbers and variation in fish species [22]. ZnO NPs have previously been shown to cause lipid peroxidation in the livers and hearts of African catfish (*Clarias gariepinus*) [23], while an increase in the levels of intracellular reactive oxygen species (ROS) was observed in zebrafish embryos exposed to ZnO NPs, resulting in toxic effects [24].

Asian seabass, *Lates calcarifer* (Bloch, 1790), known as barramundi or giant perch in Asia, is a catadromous species. Adult Asian seabass released in inland waters migrate to coastal spawning grounds, and those released at these spawning grounds return to inland areas [25,26,27]. Asian seabass is a commercially important fish species found in the tropics and subtropics. This species is essential for food production and has high economic value. Thailand is a major producer of both fry and adult fish that are in demand domestically and contribute to national and international exports [28,29]. Owing to the intensive cultivation system and high-density commercial aquaculture, Thailand’s production of Asian seabass has reached 45,000 tons, which is valued at approximately 150 million USD [30]. Recently, in Thailand, there has been a shift from cage culture systems in river mouths and coastal areas to earthen ponds in low-salinity areas of the central and eastern parts of Thailand, especially Chachoengsao, Chonburi, Samut Prakan and Samut Sakhon Provinces [30]. This transition aims to avoid disease outbreaks caused by marine pathogenic bacteria [31,32,33] and improve production via fish growth, the food conversion ratio and the benefit-cost ratio [34,35]. While these new cultured areas are highly suitable for Asian seabass aquaculture, they are suspected to be near areas that are at a high risk of contamination from various industrial chemicals and pollutants.

This study highlights the potentially significant effects of ZnO NPs on the growth and immune response of Asian seabass. The information obtained from this study is crucial for investigating immunotoxicology in economic fish species, particularly in Asian seabass farming areas in Thailand. These areas, currently cultivating fish in low-salinity environments, are affected by discharge water that may carry chemicals from both community and industrial sectors. This water may be contaminated with ZnO NPs, which are widely used in various applications. These results can be used as crucial guidelines not only for investigating the toxicological impacts of ZnO NPs on Asian seabass and the environments surrounding culture areas but also for indicating the human biosecurity risks to consumers.

## 2. Materials and Methods

### 2.1. Experimental Fish

One thousand Asian seabass (*Lates calcarifer*, Bloch) fingerlings, with average weights and total lengths of 6.68 ± 1.43 g and 8.57 ± 0.76 cm, respectively, were purchased from a nursery farm located in Chachoengsao Province, Thailand. The fish were carefully transported to the Center of Excellence in Aquatic Animal Health Management (CE-AAHM), Faculty of Fisheries, Kasetsart University. Acclimatization was conducted in a 1000 L fiberglass tank containing 5 ppt seawater for 1 week. Then, 500 fish were further acclimatized in a separate 1000 L fiberglass tank containing 5 ppt seawater for 1 week to reduce the stocking density. During this period, the fish were fed commercial feed C-5003 (Uni-President, Di An, Vietnam) at 3% of their body weight, and 20% of the water was exchanged every 2 days to remove excessive waste. Water quality parameters, which consisted of temperature, salinity, pH, dissolved oxygen (DO), total ammonia, nitrite, nitrate, alkalinity and hardness, were monitored as previously described [36]. Finally, 12 glass tanks (40 × 80 × 50 cm^3^), each containing 100 L of freshwater and 5 ppt seawater, were used to raise 20 fingerlings per tank under the same conditions as those described above.

### 2.2. Experimental Design

At the beginning of the experiment, ZnO NPs (Sigma-Aldrich, Saint Louis, MO, USA) were dissolved in distilled water to a concentration of 10,000 mg/L (ppm) in a 1000 L volumetric flask and added to the treatment tanks. Three different concentrations were prepared for tanks 4, 5 and 6; 7, 8 and 9; and 10, 11 and 12, corresponding to concentrations of 1, 5 and 50 ppm, respectively, with 3 replicates for each concentration. Tanks 1–3, which did not contain ZnO NPs, served as the control group. During the experiment, 20% of the water in each tank was exchanged every 2 days, and ZnO NPs were added in weeks 2 and 3. During these periods, water quality parameters were monitored with the same methods as indicated above.

### 2.3. Growth Parameters, Mortality and Water Quality Parameters

During the 8 weeks of the experimental trials, the amount of fish feed and weight were recorded at the start of the experiment and at weeks 1, 2, 4, 6 and 8. Thereafter, growth performance was assessed for the specific growth rate (SGR), feed conversion ratio (FCR) and average daily gain (ADG) every 2 weeks, which started from WAE 4, according to the methods described by Mukhopadhyay and Rout (1996) [37]. Additionally, water quality parameters were analyzed via standard methods as described above [36]. The number of moribund or dead fish was recorded daily.

### 2.4. Hematological Analysis

Blood samples of 1 mL were collected from 1 fish in each tank at 0, 1, 2, 4, 6 and 8 weeks using a 1 mL disposal syringe without anticoagulant. We stained 5 μL of blood with 995 μL of Natt–Herricks solution to measure the complete blood count, and 15 μL was used to analyze the hematocrit according to previously described methods [38]. The remaining part of the obtained blood sample was incubated at room temperature (RT) for 2 h and further centrifuged at 4500× *g* for 10 min (min). The separated serum was collected for an analysis of serum immune parameters.

### 2.5. Immune Parameters in Serum

#### 2.5.1. Nitroblue Tetrazolium (NBT) Dye Reduction

NBT dye reduction was used to quantify the superoxide anion. This process was carried out on the basis of the formazan production determination methods described by Segal and Levi (1975) [39]. At different time points, 175 µL of serum from fish in each treatment group at different time points was distributed in a 96-well plate in triplicate, and 25 µL of NBT solution (1 mg/mL) (Sigma-Aldrich, Saint Louis, MO, USA) was carefully added. The plate was then incubated in a dark box for 2 h. After the supernatant was removed, 175 μL of absolute methanol was added for incubation in the dark for 5 min. The methanol was then aspirated, and the plates were washed twice with 175 μL of 70% methanol. Furthermore, 125 µL of 2 N KOH and 150 µL of dimethyl sulfoxide (DMSO) (Fisher Chemical™, Pittsburgh, PA, USA) were added, and the plates were incubated at RT for 5 min. The absorbance of the solution was measured with a microplate reader (iMark™ Microplate Absorbance Reader, Bio-Rad, Hercules, CA, USA) at an optical density (OD) of 650 nm, and distilled water was used as a blank.

#### 2.5.2. Lysozyme Activity Assay

The lysozyme activity was measured according to a method adapted from Parry et al. (1965) [40]. *Micrococcus lysodeikticus* (Sigma) was prepared at a concentration of 0.2 mg/mL in sodium phosphate buffer with a pH of 6. We added 10 µL of fish serum to a flat-bottomed microtiter plate, and then 250 μL of *M. lysodeikticus* solution was added to each well. The decrease in absorbance at 540 nm was measured via an enzyme-linked immunosorbent assay (ELISA) reader (iMark™ Microplate Absorbance Reader) after incubation at room temperature for 0.5 to 6.5 min. The units of lysozyme activity were calculated. One unit corresponds to the amount of enzyme that reduces the absorbance by 0.001 per minute.

#### 2.5.3. Bactericidal Activity (BA)

*Streptococcus iniae* AAHM001 was grown in 10 mL of tryptic soy broth (TSB, Merck, Rahway, NJ, USA) at 37 °C for 24 h and centrifuged at 4500× *g* for 10 min. A bacterial suspension at a concentration of 1 × 10^9^ CFU/mL was prepared in PBS (pH 7.4) at an absorbance of 1.0 at 600 nm via the above ELISA reader and further diluted to obtain a final concentration of 1 × 10^5^ CFU/mL. The bactericidal activity was analyzed via a method adapted from the modified methods of Aly et al. (2008) [41], which involved incubating serum with *S. iniae* (1 × 10^5^ CFU/mL) at 37 °C for 2 h, spreading it onto tryptic soy agar (TSA, Merck, USA) and incubating it at 37 °C for 24 h. *S. iniae* solution (1 × 10^5^ CFU/mL) at 0 h was similarly prepared and served as a control (T_0_). The remaining bacterial colonies at 24 h (T_24_) and the initial concentration (T_0_) were counted, and the BA concentration was calculated via the following formula: %BA = [(T_0_ − T_24_)/T_0_] × 100. The bactericidal activity at different intervals was relatively calculated against the BA on day 0.

### 2.6. Zn Analysis in Fish Tissues

Approximately 1 g of wet tissue from the gills, gut, kidneys, liver, muscles and skin of the sampled fish was first digested in Teflon vessels in 10 mL concentrated HNO_3_ at 160 °C for 1 h via a Teflon beaker equipped with a microwave device following a protocol described elsewhere [42]. The solution was carefully filtered with Whatman No. 4 paper, and the volume of the obtained liquid solution was further adjusted to 50 mL with distilled deionized water. The Zn concentration was analyzed with a PerkinElmer (AAnalyst 200) Flame Atomic Absorption Spectrophotometry (AAS) system (PerkinElmer, Waltham, MA, USA), following the manufacturer’s instructions.

### 2.7. Gene Expression Analysis of Immune-Related Genes

#### 2.7.1. Total RNA Extraction and cDNA Synthesis

The anterior kidneys of the fish in each group were collected at different time intervals and quickly fixed in TRIzol reagent (Gibco BRL, Gaithersburg, MD, USA). The tissue samples were homogenized via a FastPrep^®^ homogenizer (MP Biomedicals, Irvine, CA, USA) for 40 s, followed by phase separation with chloroform, as recommended by the manufacturer’s protocol, to extract total RNA. The total RNA pellet was dried and quantified via a NanoDrop spectrophotometer (NanoDrop 2000, Thermo Scientific, Waltham, MA, USA) and adjusted to a final concentration of 1 µg/μL. A Revert Aid First Strand cDNA Synthesis Kit (Fermentas, Waltham, MA, USA) was used to convert mRNA to complementary DNA (cDNA) according to the manufacturer’s protocol.

#### 2.7.2. Quantitative Real-Time PCR (qRT-PCR) Analysis

One microgram per microliter of the first-strand cDNA that was synthesized as described above was subjected to qRT-PCR analysis via Brilliant II SYBR^®^ Green QPCR Master Mix (Stratagene, La Jolla, CA, USA) and specific primers for both the innate and adaptive immune responses of Asian seabass, including α2 macroglobulin (*α2m*), complement 3 (*c3*), CC chemokine (*cc*), cluster of differentiation 4 and 8 (*cd4* and *cd8*), C-type lectin (*C-lec*), dendritic cell-specific transcript (*dc*), hepcidin (*hep*), heat-shock proteins 70 and 90 (*hsp70* and *Hsp90*), T-cell receptor α (*tcrα*), lysozyme (*lys*), immunoglobulin M heavy chain (*igmh)* and major histocompatibility complex class IIα molecules (*mhc II*) genes, which were designed from a transcriptomic library (ASM164080v1) (Supplementary Materials Table S1). The relative expression of each gene was normalized to *β-actin* expression levels via specific primers for Asian seabass. The real-time PCR conditions were as follows: 1 cycle of 95 °C for 10 min, followed by 40 cycles of 95 °C for 30 s, 55 °C for 30 s and 72 °C for 1 min and finally 1 cycle of 95 °C for 1 min, 55 °C for 30 s and 95 °C for 30 s. The obtained threshold cycle (C_T_) was recorded to further calculate the expression levels of the immune-related genes of the tested Asian seabass via the 2^−∆∆CT^ method [43]. 

### 2.8. Histopathological Analysis

The gills, liver, head kidney, kidney, spleen and intestine tissues were dissected and stored in 10% neutral-buffered formalin for the first 24 h, after which 70% ethanol was added. The tissues were then automatically processed and embedded in paraffin wax. A YD-315 rotary microtome (Jinhua YIDI Medical Appliance Co., Ltd, Zhejiang, China) was used to cut the sections, which were 5 μm thick. The obtained sections were carefully stained with hematoxylin and eosin (H&E) and mounted onto glass slides. All prepared permanent slides were examined for histopathological alterations under a light microscope (Nikon Eclipse E 800, Melville, NY, USA) [38].

### 2.9. Data and Statistical Analysis

The growth rates, Zn concentrations and immune gene expression levels were analyzed via one-way analysis of variance (ANOVA) following Duncan’s new multiple range test (DMRT) via the Statistical Package for Social Sciences (IBM SPSS statistic version 26). Student’s *t*-test was used to compare the means of each parameter between week 0 and other weeks. All the data were plotted via GraphPad Prism V 9.0 software (GraphPad Software, San Diego, CA, USA), and the results are presented as the means ± SDs. Differences among the tested groups were considered significant if the *p* value was <0.05.

## 3. Results

### 3.1. Water Quality Analysis

During the experimental treatments, temperature, salinity, pH, dissolved oxygen (DO), ammonia, nitrite, nitrate, alkalinity and hardness were detected in ranges that were suitable for normal growth of fish, i.e., 27.17 ± 2.64–28.89 ± 1.96 °C, 4.89 ± 0.68–5.37 ± 0.45 ppt, 7.65 ± 0.54–8.96 ± 1.42, 6.00 ± 0.12–7.44 ± 0.75 mg/L, 0.232 ± 0.064–0.581 ± 0.086 mg NH_3_-N/L, 0.036 ± 0.005–0.219 ± 0.002 mg NO_2_-N/L, 0.125 ± 0.065–1.013 ± 0.045 mg NO_3_-N/L, 40.0 ± 3.46–87.0 ± 4.46 mg/L as CaCO_3_ and 24.0 ± 2.44–76.0 ± 5.53 mg/L as CaCO_3_, respectively (Supplemental Materials Figure S1A–E).

### 3.2. Fish Growth and Mortality

**Figure 1:**
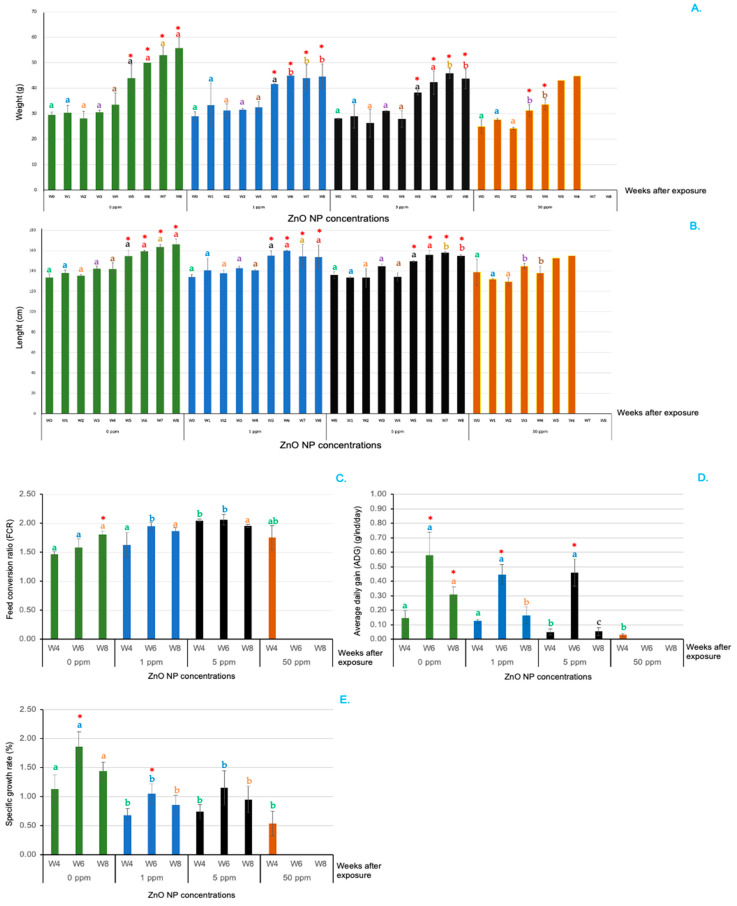
Growth parameters of Asian seabass exposed to 0, 1, 5 and 50 ppm ZnO NPs at different time intervals. Weight (**A**), length (**B**), FCR (**C**), ADG (**D**) and SPR (**E**). Red asterisks (*) indicate significant differences in each parameter between WAE 0 and other WAE (*p* < 0.05). Different letters at each WAE for different ZnO NP concentrations indicate significant differences (*p* < 0.05).

The effects of ZnO NPs on growth parameters were noted 5–8 weeks after exposure (WAE). The weight and length of the fish treated with 1, 5 and 50 ppm ZnO NPs were significantly lower than those of the control fish from 6 to 8 WAE (Figure 1A,B). The FCR of the control group was significantly lower than those of all the ZnO NP-treated groups from 4 to 8 WAE (Figure 1C). Compared with those of the control group, the ADG and SDG of all the ZnO NP-treated fish were significantly lower during the same period (Figure 1D,E).

**Figure 2:**
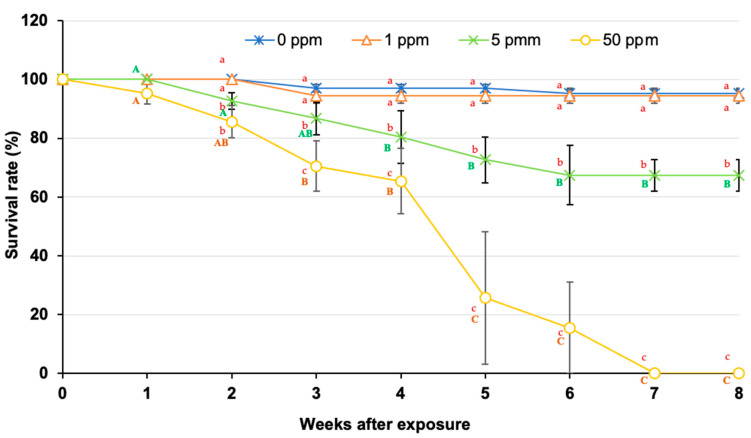
Survival rates (%) of Asian seabass exposed to 0, 1, 5 and 50 ppm ZnO NPs at different WAE. Different lowercase letters at each WAE for different ZnO NP concentrations indicate significant differences (*p* < 0.05). Different uppercase letters indicate significant differences among exposure periods within each treatment (*p* < 0.05).

Interestingly, the fish exposed to 50 ppm ZnO NPs began to die at 1 WAE, and mortality rates gradually increased from 2 to 4 WAE (Figure 2). Rapid mortality was observed from 4 to 6 WAE and reached 100% at 7 WAE. Notably, at 5 and 6 WAE, two of the three replicates resulted in 100% mortality, which strongly affected growth parameter investigations and statistical analyses during these periods (Figure 1). Five ppm ZnO NPs first induced mortality at 2 WAE, gradually increased to 32.55 ± 10.14% at 6 WAE and then stabilized at this level of mortality until 8 WAE (Figure 2). The abnormalities of moribund fish, such as dark bodies, exophthalmia, anorexia and lethargy, were always observed in these two groups. The initial mortality of the fish exposed to 1 ppm ZnO NPs was 5.55 ± 2.55% at 2 WAE, which was unchanged until 8 WAE. Lower mortality rates were observed in the control group during all the experimental periods (Figure 2). Significant differences in the mortality of the control and 1 ppm ZnO NP-treated groups and the 5 and 50 ppm ZnO NP groups were first reported at 2 WAE and at all time points until the end of the experiment at 8 WAE (*p* < 0.05) (Figure 2). Additionally, significant differences in fish mortality within groups were observed only in the 5 and 50 ppm ZnO NP groups.

Since 50 ppm ZnO NPs strongly affected fish mortality from 5 to 6 WAE, most parameters had to be ignored from 5 to 8 WAE, except for the expression of immune-related genes at 6 WAE, which was carried out on the head kidney tissues of the moribund fish.

### 3.3. Hematological Analyses

**Figure 3:**
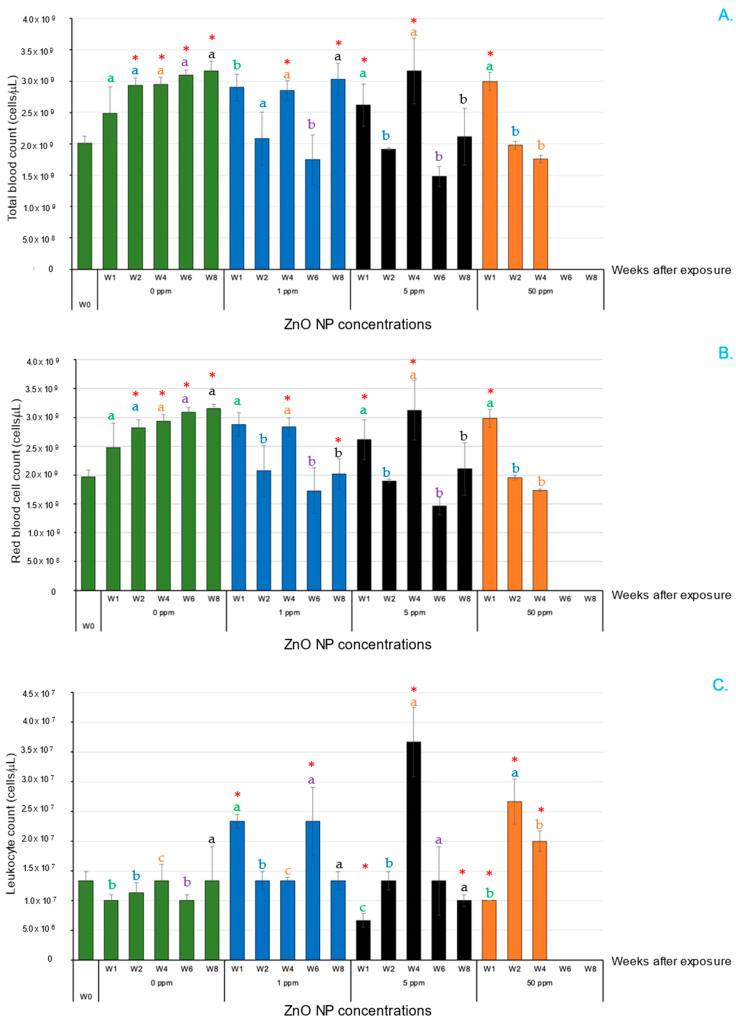

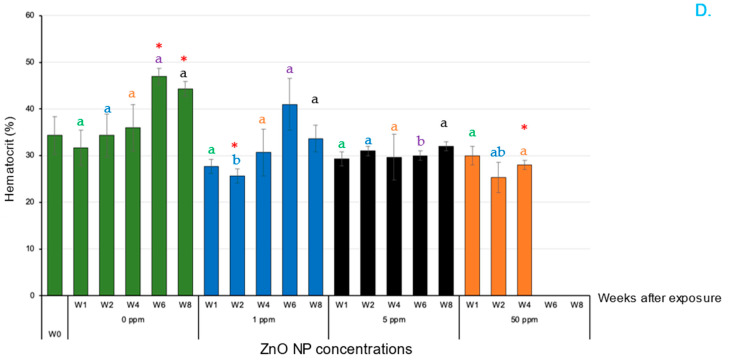
Hematological analysis of Asian seabass exposed to 0, 1, 5 and 50 ppm ZnO NPs at different WAE. Total blood count (**A**), red blood cell count (**B**), leukocyte count (**C**) and hematocrit (**D**). Red asterisks (*) indicate significant differences in each parameter between 0 WAE and other WAE (*p* < 0.05). Different letters at each WAE for different ZnO NP concentrations indicate significant differences (*p* < 0.05).

Compared with the control, exposure to 1, 5 and 50 ppm ZnO NPs severely affected the total blood count (TBC), red blood cell (RBC) count, leukocyte count (LC) and hematocrit (Hct) (Figure 3A–D). Unexpectedly, the TBC, RBC count and LC of the fish treated with ZnO NPs at these concentrations were significantly greater at 1 WAE than they were in the control group (Figure 3A–C). These parameters were inconsistently regulated from 2 to 8 WAE, but at 2 and 4 WAE, the TBC and RBC counts of the control were significantly greater than those of the 50 ppm ZnO NP-treated group (*p* < 0.05), whereas the LC of the 50 ppm ZnO NP-treated groups at 2 and 4 WAE was significantly greater than that of the control in these two periods (*p* < 0.05) (Figure 3C). Interestingly, the Hct of the control fish at 6 and 8 WAE was greater than that of all the ZnO NP-treated groups at almost all time intervals (Figure 3D).

### 3.4. Innate Immune Parameters in Serum

**Figure 4:**
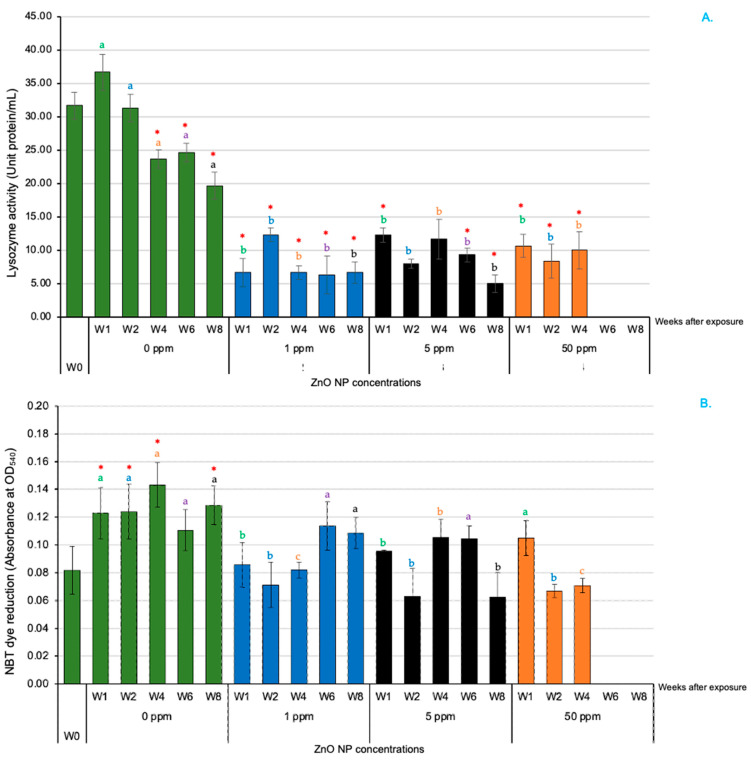

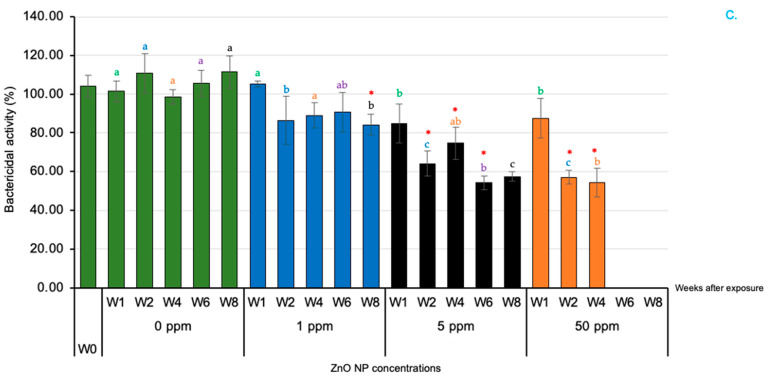
Serum innate immune parameters of Asian seabass exposed to 0, 1, 5 and 50 ppm ZnO NPs at different WAE. Lysozyme activity (**A**), NBT dye reaction (**B**) and relative bactericidal activity (**C**). Red asterisks (*) indicate significant differences in each parameter between 0 WAE and other WAE (*p* < 0.05). Different letters at each WAE for different ZnO NP concentrations indicate significant differences (*p* < 0.05).

For lysozyme activity (LA), the control-group fish exhibited significantly higher levels than those in all ZnO NP-treated groups at all time intervals (*p* < 0.05) (Figure 4A). The NBT dye reaction showed that the control-group fish had a significantly greater value than that of the fish treated with 1 ppm ZnO NPs from 1 to 4 WAE (*p* < 0.05) (Figure 4B). In terms of relative bactericidal activity (RBA), all the ZnO NP-treated groups showed a decrease in RBA proportional to the ZnO NP concentration, with the 5 and 50 ppm ZnO NP groups showing a significantly lower RBA compared with that of the control group at all WAE (*p* < 0.05) (Figure 4C).

### 3.5. Zn Concentration Measurement in Tissues

**Figure 5:**
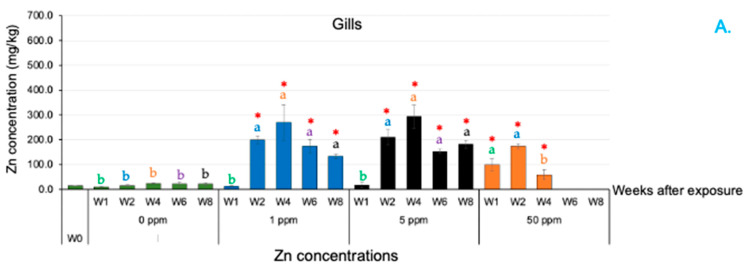

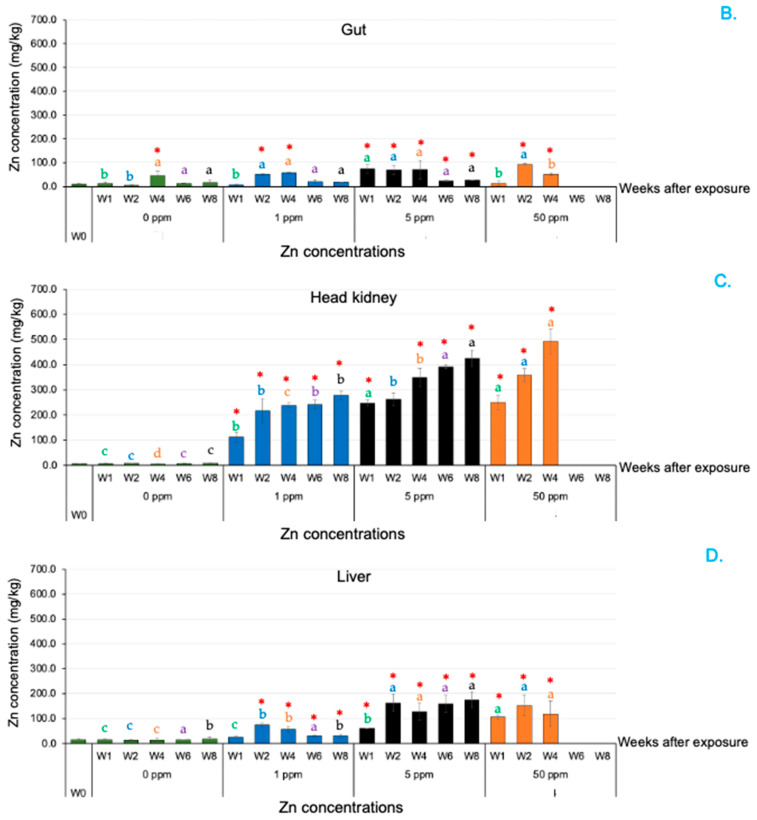

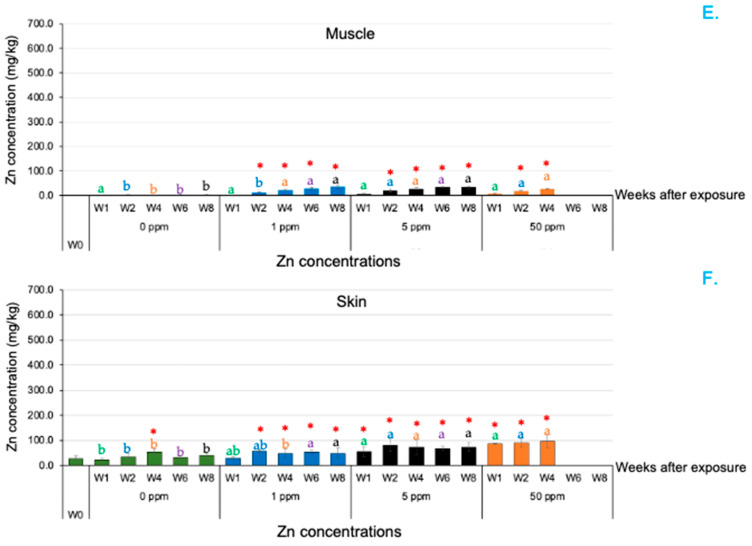
Detection of Zn concentrations in various tissues of Asian seabass exposed to 0, 1, 5 and 50 ppm ZnO NPs at different time intervals. Gills (**A**), gut (**B**), head kidney (**C**), liver (**D**), muscle (**E**) and skin (**F**). Red asterisks (*) indicate significant differences in each parameter between 0 WAE and other WAE (*p* < 0.05). Different letters at each WAE for different ZnO NP concentrations indicate significant differences (*p* < 0.05).

Zn ion concentrations were detected during the ZnO NP treatments. Overall, Zn accumulation was clearly observed in the head kidney, gills, liver, skin and muscle in all the ZnO NP-treated groups (Figure 5A–F). Zn concentrations in the head kidney of the fish in all the treated groups increased significantly from 0 to 8 WAE in the 1 and 5 ppm ZnO NP treatment groups and from 0 to 4 WAE in the 50 ppm ZnO NP-treated group (Figure 5C). At 8 WAE, the Zn concentration in the control group was 6.11 ± 1.49 mg/kg, while those in the 1 and 5 ppm ZnO NP treatment groups were 277.42 ± 18.12 and 424.25 ± 31.69 mg/kg, respectively. The maximum concentration of Zn was noted in the 50 ppm ZnO NP treatment group at 4 WAE, with a value of 492.63 ± 50.15 mg/kg.

### 3.6. qRT-PCR Analysis of the Expression of Immune-Related Genes

**Figure 6:**
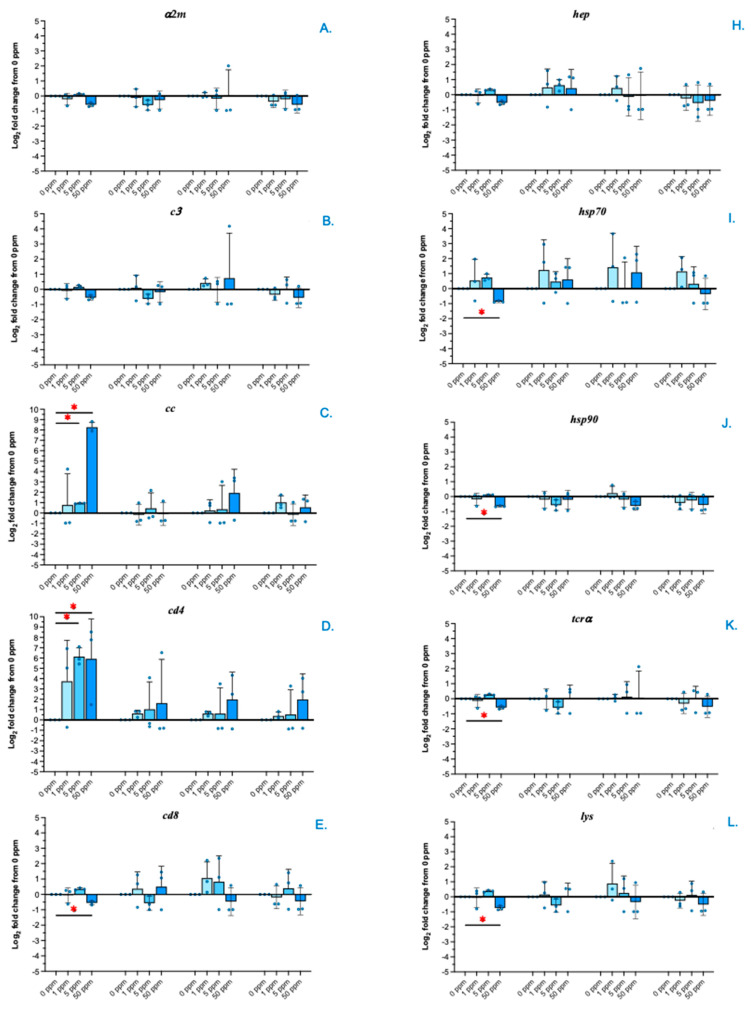

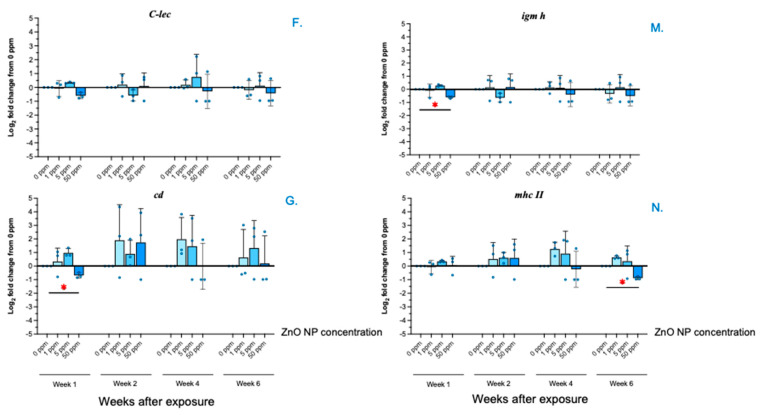
qRT-PCR analysis of immune-related genes in Asian seabass exposed to 0, 1, 5 and 50 ppm ZnO NPs at 1, 2, 4 and 6 WAE. α2 macroglobulin (*α2m*) (**A**), complement 3 (*c3*) (**B**), CC chemokine (*cc*) (**C**), cluster of differentiation 4 and 8 (*cd4* and *cd8*) (**D**,**E**), C-type lectin (*C-lec*) (**F**), dendritic cell-specific transcript (*dc*) (**G**), hepcidin (*hep*) (**H**), heat-shock protein 70 and 90 (*hsp70* and *hsp90*) (**I**,**J**), T-cell receptor α (*tcrα*) (**K**), lysozyme (*lys*) (**L**), immunoglobulin M heavy chain (*igmh)* (**M**) and major histocompatibility complex class IIα (*mhc II*) (**N**) genes. Red asterisks (*) indicate significant differences in each parameter between WAE 0 and other WAE (*p* < 0.05).

The expression of immune-related genes involved in both the innate and adaptive immune responses was investigated in the head kidneys of all the treatment groups (Figure 6A–N). Interestingly, at 1 WAE, the *cc* and *cd4* genes in the 5 and 50 ppm ZnO NP treatment groups were significantly upregulated compared with those in the control and 1 ppm ZnO NP-treated groups (*p* < 0.05) (Figure 6C,D). On the other hand, the *hsp70*, *hsp90*, *tcrα*, *cd8*, *lys*, *igmh* and *DC* genes were significantly downregulated only in the 50 ppm ZnO NP-treated group at 1 WAE (Figure 6I, Figure 6J, Figure 6K, Figure 6E, Figure 6L, Figure 6M and Figure 6G, respectively). Furthermore, compared with those in the other groups, the *mhc II* gene in fish treated with 50 ppm ZnO NPs was also downregulated (*p* < 0.05) (Figure 6N).

### 3.7. Histopathological Alterations

**Figure 7:**
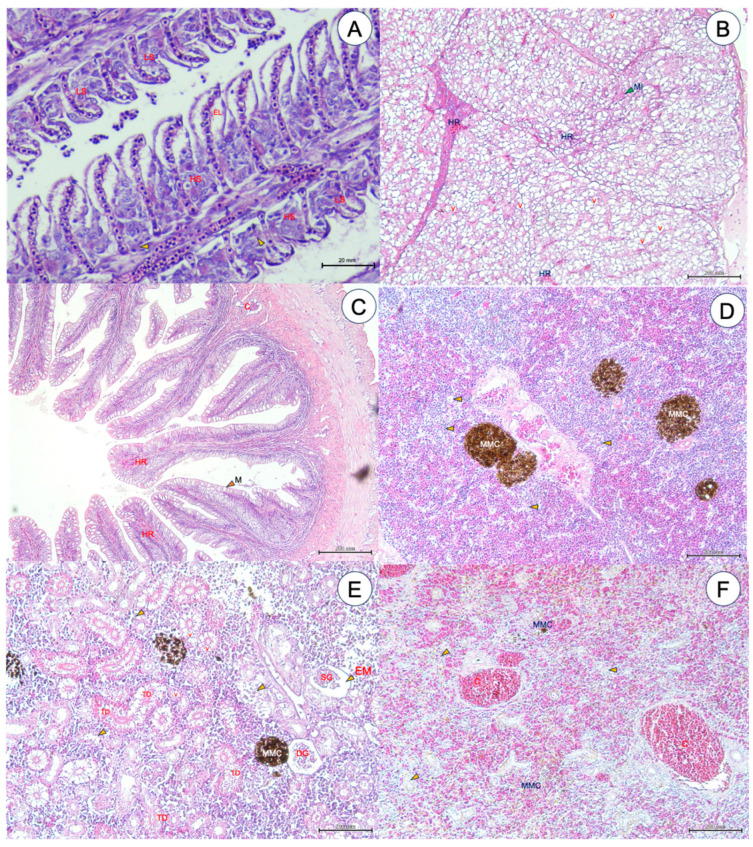
Histopathological analysis of the gills (**A**), liver (**B**), intestine (**C**), head kidney (**D**), trunk kidney (**E**) and spleen (**F**) of Asian seabass exposed to 5 and 50 ppm ZnO NPs. EL; epithelium lifting, LS; lamellar shortening, HS; hyperplasia, MI; mononuclear cell infiltration, HR; hemorrhage, v; vacuolations, C; congestion, M; mucus cells, MMC; melanomacrophage centers, TD; renal tubular deformations, EM; edema, SG; shrinkage glomerulus, DG; degeneration of glomerulus. Necrotic degeneration is indicated by yellow arrow points and mm = micrometer.

Histopathological changes in the gill, liver, intestine, head kidney, trunk kidney and spleen tissues were primarily observed in most of the moribund fish exposed to 5 and 50 ppm ZnO NPs at WAE 5–8 (Figure 7A–F). In summary, in the gills, epithelium lifting (EL), lamellar shortening (LS), necrotic degeneration and hyperplasia (HS) were normally observed (Figure 7A). In the liver, mononuclear cell infiltration (MI), hemorrhage and fatty changes or hepatocellular vacuolations (v) were clearly observed (Figure 7B). In the intestine, hemorrhage (HR) in lamina propria and congestion (C) were observed (Figure 7C). In the head kidney, melanomacrophage centers (MMC) and many areas of necrotic degeneration were clearly recorded (Figure 7D). Histopathological alterations in MMC, renal tubular deformations (TD), necrosis and cytoplasmic vacuolations (v), edema (EM) and shrinkage (SG) or degeneration of glomerulus (DG) were widely detected in the trunk kidney (Figure 7E). Finally, necrotic degeneration, MMC and huge congestion (C) were clearly observed in the fish spleen (Figure 7F).

## 4. Discussion

ZnO NPs, which are widely used in various activities and industries, are known to have detrimental effects on aquatic environments, especially in the aquaculture industry. To date, Asian seabass aquaculture has undergone significant change from cage culture systems in coastal and river mouth areas to the earthen ponds in low-salinity areas of the central and eastern parts of Thailand. The aim of this transition is to avoid harmful diseases caused by pathogenic bacteria, such as *Vibrio* spp., *Streptococcus* spp., *Flavobacterium* spp. and *Tenacibacculum* spp. [31,32,33], and increase growth rates and production [34,35]. ZnO-NPs, with a global production of approximately 1.2 million tons [44], pose a high risk and can easily contaminate aquatic ecosystems, mainly through discharged wastewater [45]. Therefore, the toxicological effects of ZnO NPs in Asian seabass are worth investigating to generate crucial knowledge that is important for revealing this obscure information.

After the Asian seabass groups were subjected to prolonged exposure to ZnO NPs at concentrations of 1, 5 and 50 ppm for eight weeks, with treatments applied weekly for three consecutive weeks at weeks 0, 1 and 2, the results revealed that exposure to 1, 5 and 50 ppm had negative effects on all growth parameters and hematological and innate immunity indices in the serum. Additionally, 50 ppm ZnO NPs significantly suppressed the expression levels of both innate and adaptive immune-related genes at 1 WAE. This information is in accordance with the mortality rates of the fish, in which 50 ppm ZnO NPs caused 100% mortality at 6 WAE, whereas the 5 ppm treatment resulted in 32.55 ± 10.14% mortality at the end of the experiment. However, the mortality rates of the 1 ppm exposure group were low, significantly lower than those of 5 and 50 ppm, and did not differ from those of the control group. These findings indicate that 5 and 50 ppm ZnO NPs can have severe toxic effects on the growth and health of Asian seabass. Even though 1 ppm ZnO NPs can result in lower mortality rates, their impacts on the growth and health status of Asian seabass cannot be disregarded. Based on our results, a longer exposure period with a low concentration of ZnO NPs at 1 ppm from WAE 2 or 4 seriously caused impairments of both hematology and serum immune parameters, while concentrations of Zn concisely showed high accumulated trends in both the gill and head kidney tissues. These findings suggest that, even with a low concentration of 1 ppm for a long duration, ZnO NP exposure can impair growth, affect health status and increase susceptibility to various pathogens, which may lead to severe disease and production losses in the culture system.

Previously, ZnO NPs were classified as “extremely toxic”, with a median lethal concentration (LC_50_) of less than 0.1  mg/L in aquatic organisms [46]. However, on the basis of the much-updated information in the current research, the toxicity of ZnO NPs to aquatic organisms varies considerably across tested species and experimental conditions, such as temperature, pH, timing, exposure periods and other factors [47]. Additionally, the toxic mechanisms of ZnO NPs in various aquatic organisms include the release of Zn^2+^ ions, which strongly interrupt the homeostasis of organisms by passing through the cell membrane and combining with mitochondria. This can further induce the apoptotic pathway [48], membrane internalization and disorganization, which induce direct particle–cell contact [49,50], and the generation of ROS, which consequently induce oxidative stress and trigger the autophagy process during this oxidative stress process [51,52]. The application of ZnO NPs has a high potential for significantly damaging the immune system and inhibiting Na^+^ and K^+^ ATPase activity, resulting in increased serum Ca^2+^ and Cl^-^ levels in the gills, consequently impairing osmoregulatory activity and toxicity in Nile tilapia [53]. Moreover, in ZnO NP-treated zebrafish, the target substance strongly inhibited the normal development and growth of the experimental animals, and the cell cycle was severely affected by other life activities. Additionally, ZnO NPs were found to strongly disturb the normal activities of individual organisms, especially differentiation, cell division and proliferation, and the functions of intracellular transfer and DNA binding were ultimately distressed [54].

In previous toxicological studies of ZnO NPs in various fish species, median lethal concentrations at 96 h (96 h-LC_50_) [55] were used, with values ranging from 2.37 ppm in Asian stinging catfish (*Heteropneus fossilis*) [56] to 31.15 ppm in rohu (*Labeo rohita*) [57]. On the basis of these studies, two major mechanisms of ZnO NP toxicity involving the impairment of physiological and biochemical factors in four major parts that cause severe mortality in fish, including the gills, blood, intestines and liver, can be summarized [55].

In our study, the toxicological effects of ZnO NPs on histopathological changes and serum innate immune parameters in treated fish were clearly observed. ZnO NP levels increased in a concentration- and time-dependent manner in the organs, similar to the levels reported in Nile tilapia treated with 1 and 10 mg/L ZnO NPs for 14 days [53], and decreased various hematological factors, such as red blood cells, hematocrit and hemoglobin [4]. Furthermore, these applications significantly affect antioxidant defense system biomarkers, including superoxide dismutase, catalase and glutathione levels, depending on particle size, exposure time and concentration [58]. However, the most influential organs were the intestine, liver, kidney and gills, which differed from our study. In the present case, the head kidney, gills, liver and skin were the most affected organs, suggesting that the Asian seabass may have a physiological defense capacity that differs from that of freshwater fish.

The strong suppression of both innate and adaptive immune gene expression, cellular stress involving *hsp70* and *hsp90* genes and serum innate immune factors in the early exposure stages after ZnO NP exposure cannot be ignored and may have strongly affected the Asian seabass in our study. In fish, the effects of ZnO NPs on these parameters are unclear. However, innate immunity of nematode worms (*Caenorhabditis elegans*) was strongly suppressed by ZnO NPs via inhibition of SKN-1/Nrf and the p38 MAPK signaling pathway [59], which plays crucial roles in environmental oxidative stress signaling and pathogenic stress [60]. In the Raw 264.7 mouse macrophage cell line, ZnO-NPs seriously caused immunotoxicity by suppressing innate immunity and T helper-1 cytokines, and inflammation in the serum was significantly suppressed [61]. Combined with findings from previous studies, our findings suggest that exposure of aquatic animals to ZnO NPs may severely induce oxidative stress in crucial organs, such as the liver, head kidney, intestine and spleen; suppress biochemical parameters in the serum; and impair hematological components and immune responses of both the innate and adaptive immune systems [53,55,58]. The immune-suppressing activity of ZnO NPs was also observed in common carp (*Cyprinus carpio*) through their ability to reduce lysozyme and alternative complement activity (ACH_50_), alkaline phosphatase activity, protease activity and total immunoglobulin (total Ig) content [3]. Furthermore, the head kidney, which is an important primary lymphoid organ of the fish immune system, should be considered one of the major targets of ZnO NPs in addition to the gills, blood, intestines and liver, as noted in a previous review [55]. Based on these reports, mechanisms of regulation in immune systems underlying the immunotoxicity of ZnO-NPs are largely unclear.

Influencing factors, especially water quality parameters, have been reported to affect ZnO NP toxicity. ZnO NPs exhibited low toxicity levels when exposed to water at pH values ranging from 6.5 to 8.5, resulting from their ability to regulate the surface charge of the nanoparticles and consequently decrease the solubility of the ZnO NPs, ultimately further increasing their stability and aggregation [62]. Yung et al. (2017) [63] studied the effects of temperature and salinity on the toxicity of ZnO NPs in *Thalassiosira pseudonana* and reported that ZnO NPs at high levels of these two factors significantly induced larger volume aggregation and released low concentrations of Zn^2+^, causing less toxicity in target animals. Similarly, Lai et al. (2020) [64] reported that high salinity significantly decreased ZnO NP toxicity to *Tigriopus japonicus* copepods because high salinity could significantly increase osmotic pressure in copepods and, under low salinity conditions, effectively induce ion dissolution. Additionally, with increasing salinity from freshwater (0 ppt) to brackish water (10 ppt), ZnO NPs in the form of Zn^2+^ decreased toxicity by reducing their dissolved content, resulting in a better hatching rate of embryos and survival rate of zebrafish (*Danio rerio*) larvae [65]. With respect to this information, low-salinity conditions should be carefully considered to determine the rigid toxicity of metal oxide nanoparticles compared with high-salinity conditions [66]. Therefore, the toxicity of ZnO NPs to Asian seabass under low salinity can be considered to explain the effects of this chemical under affected culture conditions. Based on water quality parameters, salinity was set at approximately 5 ppt in all treatments, whereas the other physicochemical and chemical parameters were satisfactorily in line with the optimal levels for the normal growth of aquatic animals [67], indicating the reliable effects of the ZnO NPs in the target experimental animal in the present study.

## 5. Conclusions

Our findings support and contribute to our understanding of the toxicity of ZnO NPs for Asian seabass and provide an investigative basis for determining the environmental and social impacts of nanomaterials. Recently, the Food and Drug Administration (FDA) of the United States of America stated that ZnO NPs should be generally recognized as safe (GRAS) substances for application in humans. However, the information in our study and various reports concisely indicates that the application of ZnO NPs has deleterious effects on aquatic animals by influencing their physiological, biochemical and immunological factors. Therefore, the application of this nanomaterial product must be carried out with care, and its contaminated water discharge should be carefully considered and well-monitored to prevent negative side effects on aquatic animals, aquatic ecosystems and consumers, who are at the top levels of the ecosystem and may be indirectly affected by the contamination of ZnO NPs in aquaculture products.

## Data Availability

The data that support the findings of this study are available upon request from the corresponding authors.

## Supplementary Materials

The following supporting information can be downloaded from https://www.mdpi.com/article/10.3390/ani14182737/s1, Table S1: Primers used for the quantification of immune-related gene expression by qRT-PCR in Asian seabass (*Lates calcarifer*). Figure S1: Water quality parameters during ZnO NP treatment of Asian seabass.
