# Supplementary material for: Effects of Zinc Oxide Nanoparticles (ZnO NPs) on Growth, Immune Responses and Histopathological Alterations in Asian Seabass (Lates calcarifer, Bloch 1790) under Low-Salinity Conditions
Source: Animals (Basel). 2024 Sep 21;14(18):2737. doi: 10.3390/ani14182737 (PMC11428624; doi:10.3390/ani14182737)
Supplement: Supplementary file 1 [file animals-14-02737-s001.zip › animals-3186222-supplementary.pdf]

Supplementary Material Table S1. Primers used for quantification of immune-related gene expression by qRT-PCR of Asian seabass (*Lates calcarifer*).

Gene	Primer name	Nucleotide sequences (5'→3')	Amplicon size (bp)	Tm (ºC)	Reference
α-2-macroglobulin (*α-2m*)	*Lc_ α2M*	F: TGCTTTCTGGTTTTGCC<br>R: TGGTTGATGCCTATGTCC	136	60	ASM164080v1
Complement 3 (*c3*)	*Lc_ C3*	F: GCAATCCTCCACAACTACAG<br>R: ACTCTGACCTCCTGACGATAC	11	60	
CC chemokine (*cc*)	*Lc_ CC*	F: CCTGCCCTGTGTCAATG<br>R: TTGCGGGCCTCTTCTAG	111	60	
CD4 receptor for T-helper cell (*cd4*)	*Lc_CD4*	F: AGTGCAATGGATTGGGGTAGATAA<br>R: GTTGCAGGCTCTGTAACTTTGATT	156	60	
CD8 receptor for T-cytotoxic cell (*cd8*)	*Lc_CD8*	F: AATTCCTTCAGCAAAGCTCGTG<br>R: GTGGTTGTGGGTAGTCTTGGAT	188	60	
C-type lectin (*C-lec*)	*Lc_ CLEC*	F: AAGGGTGGTTTCAAGTAGAG<br>R: GTCAAGATGGCAAGATGTC	112	60	
Dendritic cell-specific transcript (DC-specific) (*cd*)	*LC-DCs*	F: AACAGCACACGCTCACTCAC<br>R: CGATCATGTGAGCCTTGAGA	153	60	
Hepcidin (*hep*)	*Lc_ Hep1*	F: ATTTGCATTCTGGAGAGCTCTGCC<br>R: CCATTGACATCTCTTGATGTGCCG	208	60	
Heat shock protein 70 kDa (*hsp70*)	*Lc_ Hsp70*	F: AAGGCAGAGGATGATGTC<br>R: TGCAGTCTGGTTCTTGTC	186	60	
Heat shock protein 90 kDa (*hsp90*)	*Lc_ Hsp0*	F: ACCTCCCTCACAGAATACC<br>R: CTCTTGCCATCAAACTCC	197	60	
Immunoglobulin M heavy chain (*igm H*)	*LC-IgMH3*	F: AAGGTAAACGAGGGAGCTGTTG<br>R: TGGCTCCATGCCATCTACAAGT	192	60	
Lysozyme (*lys*)	*Lc_ Lys*	F: TGCATCACACACCATGGCAA<br>R: CATCCACGTTGTCATAGGAG	401	60	
Major histocompatibility complex (*MHC*) class IIα molecules (*mhc IIα*)	*LC-MHC II*	F: TCTGAACCAACCACTGACCAGA<br>R: CTGATGACATCATCAGCTCCAG	177	60	
T-cell receptor *α* (*tcr α*)	*LC-TCR a*	F: ACCCAACCAACAGTGACAGTGT<br>R: CTCAACCTGCTGGTGATTCTGT	233	60	

ß-actin	*Lc_ß-actin*	F: CTTCACCACCACAGCCGAGA<br>R: TGCCGATGGTGATGACCTGT	157	60
